# Physical properties and biocompatibility of an injectable calcium-silicate-based root canal sealer: *in vitro* and *in vivo* study

**DOI:** 10.1186/s12903-015-0112-9

**Published:** 2015-10-21

**Authors:** Eun-Su Lim, Young-Bae Park, Young-Sun Kwon, Won-Jun Shon, Kwang-Won Lee, Kyung-San Min

**Affiliations:** Department of Conservative Dentistry, School of Dentistry and Institute of Oral Bioscience, Chonbuk National University, 567 Baekje-daero, Jeonju-si, 54896 Korea; Research Institute of Clinical Medicine of Chonbuk National University-Biomedical Research Institute of Chonbuk National University Hospital, 20 Geonji-ro, 54907 Jeonju-si, Korea; Department of Conservative Dentistry, Dental Research Institute and School of Dentistry, Seoul National University, 101 Daehak-ro, 03080 Seoul, Korea

**Keywords:** Injectable, Calcium silicate, Root canal sealer, Physical, Biological

## Abstract

**Background:**

The aim of this study was to investigate the physical properties and biological effects of an experimentally developed injectable premixed calcium-silicate root canal sealer (Endoseal) in comparison with mineral trioxide aggregate (MTA) and a resin-based sealer (AHplus).

**Methods:**

The pH, solubility, dimensional change, flow, and radiopacity of the materials were evaluated. Biocompatibility was evaluated on the basis of cell morphology and a viability test using MC3T3-E1 cells. For evaluate inflammatory reaction, the tested sealers were implanted into dorsal subcutaneous connective tissue of Sprague Dawley rats. After 7 days, the implants with the surrounding tissue were retrieved, and histological evaluation was performed.

**Results:**

Endoseal showed high alkalinity similar to that of MTA. The solubility of the tested materials was similar. The dimensional change and flow of Endoseal was significantly higher than that of other materials (*P* < 0.05). The radiopacity of Endoseal was lower than that of AHplus (*P* < 0.05). The biocompatibility was similar to those of MTA. Inflammatory reaction of Endoseal was similar with that of MTA, but lower than that of AHplus (*P* < 0.05).

**Conclusions:**

The present study indicates that Endoseal has favorable physical properties and biocompatibility. Therefore, we suggest that Endoseal has the potential to be used as a predictable root canal sealer.

## Background

**Fig. 1:**
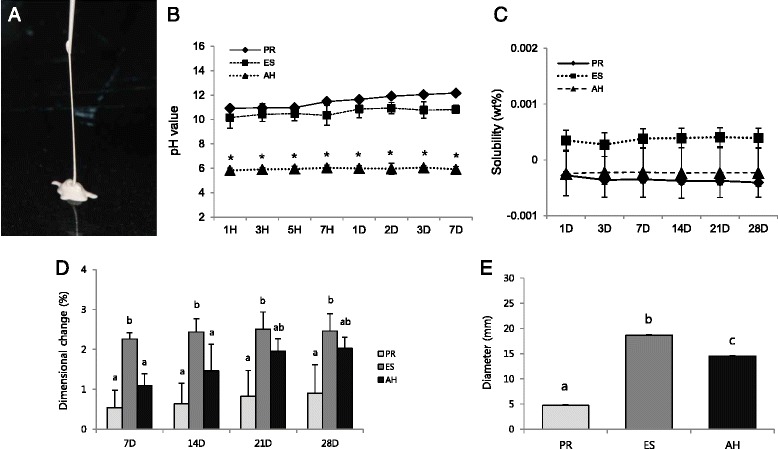
Physicochemical properties of the tested materials. **a** The injectable calcium-silicate-based root canal sealer used in this study. **b** The changes in pH value during the experimental period. Groups identified by the same symbols were not significantly different in the same gene group (*P* > 0.05). Solubility (**c**), dimensional change (**d**), and **e** flow of the tested materials. Different letters/symbols represent significant differences between the different endodontic sealers (*P* < 0.05). *PR; ProRoot, ES; Endoseal, AH; AHplus*

Endodontic sealers are used for the obturation of root canal systems in order to achieve a fluid-tight seal between the dentinal wall and core filling material throughout the entire canal [1]. A root canal sealer must demonstrate appropriate physicochemical and biological properties. Grossmann stated that an ideal root canal sealer should possess excellent sealing ability, dimensional stability, a slow setting time, insolubility, and biocompatibility [2]. There are many types of root canal sealers available in the endodontic market; resin-based sealers, zinc oxide-eugenol sealers, calcium hydroxide-containing sealers, glass ionomer-based sealers, and mineral trioxide aggregate (MTA)-based calcium-silicate sealers. All of the currently used sealer systems consist of a powder/liquid or base/catalyst, and the two components should be mixed at chairside and then applied to the root canal system. Recently, an injectable calcium-silicate-based root canal sealer (Endoseal; Maruchi, Wonju, Korea) that is preserved in an air-tight syringe and applied in the root canal by injection was developed (Fig. 1a). Interestingly, Endoseal sets slowly by itself without any mixing when exposed to air by absorbing the ambient moisture.

According to the manufacturer, this calcium-silicate cement is considered an MTA-derived material because it contains similar chemical elements as MTA. Therefore, it is expected to have favorable physical and biological effects like those of various MTA-derived materials demonstrated in previous studies [3–5]. Furthermore, many studies showed that MTA-derived root canal sealers have higher biocompatibility compared to resin-based sealers [6–9]. However, to our knowledge, there is little information regarding the self-setting calcium-silicate-based root canal sealer. Therefore, the aim of this study was to investigate the physical properties and biocompatibility of this root canal sealer in comparison with MTA (ProRoot; Dentsply, Tulsa, OK, USA) and a resin-based sealer (AHplus; Dentsply-De Trey, Konstanz, Germany).

## Methods

### Measurement of pH

The pH was measured according to the criteria used in a previously published study [10]. Specimens (1-mm thickness and 5-mm diameter) of the tested materials were prepared and allowed to set for 1 day (*n* = 3). After setting, one tablet was added to 10 mL of deionized water. Then, the pH value was measured using a pH meter (Orion 3 Star; Thermo Scientific, Singapore). The apparatus was previously calibrated with pH 7.0 and 4.0 solutions.

### Evaluation of solubility

The solubility was measured by using the method recommended by ISO 6876/2012. Samples of each material were placed in a paraffin wax mold 1.5 mm thick and 20 mm in diameter (*n* = 3). Each sample was weighed using an analytical balance, and the weight was recorded as W_1_. The samples were then immersed in tubes containing 10 mL of distilled water. Samples were removed at 1, 3, 7, and 14 days, dried with absorbent paper, and placed in a desiccator. The samples were dried to a constant weight (±0.001 g), which was recorded as W_2_. The solubility (S) was calculated using the following formula: S = (W_1_ – W_2_)/W_1_ × 100.

### Dimensional change

The dimensional change was measured by using the method recommended by ISO 6876/2012. Each material was placed into a cylindrical silicon mold with an internal diameter of 6 mm and a height of 12 mm (*n* = 5). After setting, we measured the distance between the flat ends (M1) to an accuracy of 10 μm by using a digital caliper (Absolute Digimatic, Mitutoyo, Kawasaki, Japan). The materials were then stored in distilled water at 37 ± 1 °C. After 7, 14, and 21 days, the distance (M_2_) was re-measured to an accuracy of 10 μm. The test was carried out three times, and the mean change in length was recorded as the dimensional change (D) using the following formula: D = (M_2_ – M_1_)/M_1_ × 100.

### Flow test

The flow was tested by using the method recommended by ISO 6876/2012. A total of 50 mg of sealer was placed onto a glass plate (*n* = 3). After 180 s, another glass plate was applied centrally on top of the material, to make a total mass on the plate of 120 g. Ten minutes after the application, the load was removed, and the average of the major and minor diameters of the compressed discs was measured using a digital caliper. The mean of three measurements for each sealer was taken as the flow of the material.

### Radiopacity

**Fig. 2:**
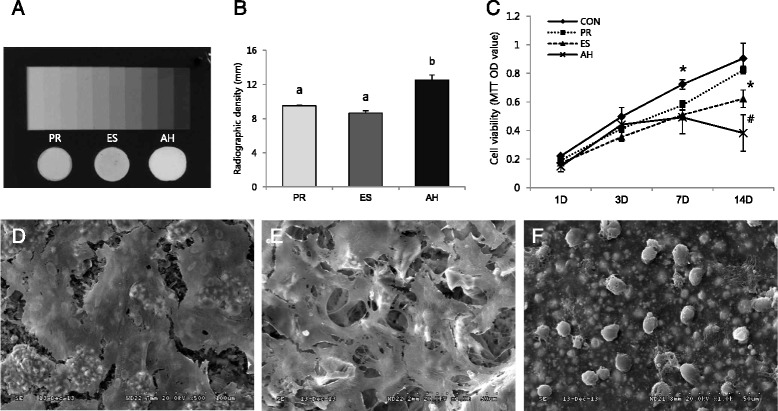
Radiopacity and biocompatibility of the tested materials. **a** Radiograph showing the radiopacity of each material and its equivalence to that of the aluminum step wedge. **b** Relative radiographic density of each material in comparison with that of a 10-step aluminum step wedge. **c** Cell viability tested by the MTT assay. **d**-**f** SEM micrographs of MC3T3-E1 cells grown on ProRoot, Endoseal, and AHplus, respectively (×1000). Different letters/symbols represent significant differences between the different materials (*P* < 0.05). *PR; ProRoot, EC; Endoseal, AH; AHplus*

The radiopacity was measured by using the method recommended by ISO 6876/2012. The specimens were placed on occlusal X-ray film (Kodak Insight, Rochester, NY, USA) along with an aluminum (99.5 % pure) step wedge with step heights ranging from 1 to 10 mm in increments of 1 mm (*n* = 5). A Kodak-2200 X-ray machine (Kodak) operating at 70 kV, 10 mA, 18 pulses/s and with a focus-sensor distance of 30 cm was used. After the films were developed, they were transformed into digital images (Fig. 2a) at a resolution of 300 dpi using a scanner. Then, the radiographic images were analyzed using a densitometer (GS-800; Bio-Rad, Hercules, CA, USA). In brief, we created a calibration curve for the aluminum step wedge, then the optical density of each specimen was expressed in terms of the equivalent thickness of the wedge in accordance with the following formula: *y* = *a*ln*x* + *b* (*y*: optical density, *x*: thickness of aluminum, ‘*a*’ and ‘*b*’: coefficients, ln: natural log value).

### Preparation of material extracts

The tested material was placed into a paraffin wax mold (1-mm thickness and 5-mm diameter). After setting, the cement was removed from the mold and stored in 10 mL of minimal essential medium-α (MEM-α; HyClone Laboratories, Logan, UT, USA) containing 10 % fetal bovine serum (FBS; HyClone Laboratories) for 3 days.

### Cell viability test

MC3T3-E1 cells were seeded in 24-well culture plates (SPL Life Sciences, Pocheon, Korea) at a density of 2 × 10^4^ cells per well and pre-incubated in growth medium for 24 h (*n* = 5). Then, the cells were treated with the prepared extracts for 1, 3, 7, and 14 days. Cell viability was measured using the 3-(4,5-dimethylthiazol-2-yl)-2,5-diphenyltetrazolium bromide (MTT) assay. Briefly, 200 μL of MTT solution (0.5 mg/ml in PBS) (Amresco, Solon, OH, USA) was added to each well, and the wells were incubated for 2 h. Subsequently, 200 μL of dimethyl sulfoxide (DMSO; Amresco) was added to each well. Reduced MTT was then measured spectrophotometrically at 540 nm in a dual-beam microtiter plate reader (SPECTROstar Nano; BMG Labtech, Ortenberg, Germany).

### Cell morphological observations using SEM

Under aseptic conditions, materials were condensed into 1 × 5-mm round wax molds. The materials were allowed to set for 24 h in a humidified incubator at 37 °C. Then, the disks were placed at the bottom of 24-well tissue culture plates (SPL Life Sciences). MC3T3-E1 cells were seeded at 1 × 10^5^ cells per well on the prepared materials. After a 72-h incubation period, the dishes were fixed with 2.5 % glutaraldehyde (Sigma-Aldrich, St. Louis, MO, USA) for 2 h. Samples were then dehydrated in increasing concentrations of ethanol (70 %, 80 %, 90 %, 95 %, and 100 %) for 20 min at each concentration and immersed in n-butyl alcohol (Junsei Chemical Co., Tokyo, Japan) for 20 min. SEM was performed using an SN-3000 system (Hitachi, Tokyo, Japan) operated at 10 kV.

### Histological evaluation of inflammatory reaction

The inflammatory reactions of animal tissue to ProRoot, Endoseal, and AHplus were evaluated (*n* = 6). The sealers were inserted into sterile polyethylene tubes approximately 10 mm height and 3 mm in inner diameter. After setting, the materials were implanted in the Sprague Dawley rats’ dorsal subcutaneous tissue. An empty tube was used as the negative control. In brief, the animals were anesthetized with 0.33 mL/100 g xylazine hydrochloride (Rompun, Bayer, Leverkusen, Germany) and 0.2 mL/100 g zolazepam (Zoletil 50; Virbac SA, Carros, France), followed by shaving of dorsal fur, disinfection, incision, and divulsion of the subcutaneous tissue to insert the testing materials. Each animal received 4 materials. The position in which each sealer was implanted was standardized. The incisions were closed using a 5–0 Vicryl suture material (Johnson & Johnson, Lenneke Marelaan, Belgium).

After 7 days, the animals were euthanized by CO_2_ inhalation. An excisional biopsy of the implant area was performed with a safety margin of 1 cm. The samples were fixed in 4 % paraformaldehyde for 24 h, and the materials were removed from the samples. Then, the samples were set in paraffin blocks, and processed for histologic analysis. Sections with a thickness of 5 μm were stained with hematoxylin-eosin. Three representative sections were examined under a light microscope by a single-blinded, calibrated examiner. Quantitative evaluations of inflammatory cells (lymphocytes and polymorphonuclear leukocytes) were made in ten separate areas of sections at ×400 magnifications. An average value for each material was obtained from the sum of cells counted in ten separate areas. Inflammatory reactions were scored and evaluated according to the criteria used in a previously published study with slight modification as follows [11]; 0, none or few inflammatory cells and no reaction; 1, <25 cells and mild reaction; 2, between 25 and 125 cells and moderate reaction; 3, ≥ 125 cells and severe reaction. These experimental procedures were approved by the Institutional Animal Care and Use Committees (IACUC) of Chonbuk National University Hospital (Jeonju, Korea).

### Statistical analysis

Statistical analysis was performed by one-way ANOVA followed by Tukey’s test for physical properties, cell viability, and gene expression assay (*P* = 0.05). For histological evaluation, the data were evaluated using one-way nonparametric Kruskal–Wallis for a 5 % significance level.

## Results

### Measurement of pH, solubility, dimensional change, flow, and radiopacity

The pH values of ProRoot and Endoseal showed high alkalinity (pH between 10 and 12), whereas AHplus showed mild acidity around pH 6 (Fig. 1b). The solubility values of the tested materials were similar throughout the experimental period (*P* > 0.05) (Fig. 1c). As shown in Fig. 1d, the dimensional change of Endoseal was significantly higher than that of the other materials at all experimental time points (*P* < 0.05). The flow of Endoseal was significantly higher than that of other materials (*P* < 0.05) (Fig. 1e). The radiopacity of AHplus was higher than those of ProRoot and Endoseal (*P* < 0.05). However, all the materials evaluated presented the minimum radiopacity required by the ISO standard (Fig. 2b).

### Biocompatibility

**Fig. 3:**
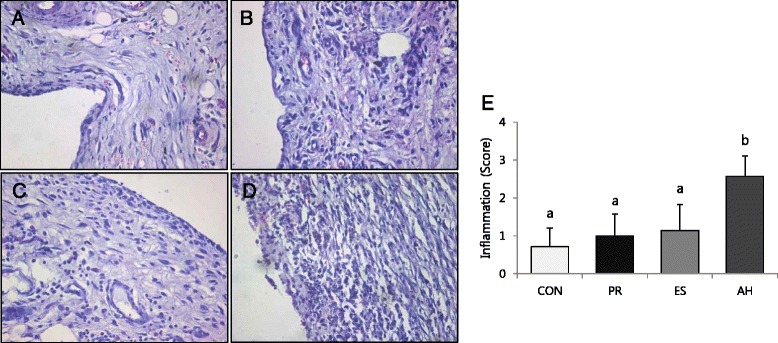
Reaction of rat subcutaneous connective tissue to the tested sealers and the control group after 7 days (H & E staining, ×100); **a** control, **b** ProRoot, **c** Endoseal, **d** AHplus. **e** Mean and standard deviation of histological scores. Different letters represent significant differences between the different materials (*P* < 0.05)

To evaluate cell viability in the presence of the material extracts, an MTT assay was performed. As shown in Fig. 2c, ProRoot showed significantly higher cell viability compared to the other groups on 14-day (*P* < 0.05). Further, the viability of Endoseal-treated cells was significantly higher than that of AHplus-treated cells on 14-day (*P* < 0.05). The cell growth and morphology on each material were evaluated by SEM. As shown in Fig. 2d and e, well-spread and flattened cells were observed in contact with the surfaces of ProRoot and Endoseal. On the contrary, round but dead cells were observed on the surface of AHplus (Fig. 2f). Moreover, in histological evaluation, inflammatory scores of ProRoot and Endoseal group were significantly lower than that of AHplus group (*P* < 0.05) (Fig. 3).

## Discussion

According to Grossman, an ideal root canal sealer should provide various physical properties [2]. Among them, we evaluated pH, solubility, dimensional change, flow and radiopacity. In our study, Endoseal showed high alkalinity (pH 10–11) similar to that of ProRoot (Fig. 1b). The base material of Endoseal is calcium-silicate with a chemical composition very similar to that of MTA. It is generally believed that MTA and its derivatives dissolve into calcium hydroxide when coming into contact with soft tissue, which results in a high pH [12]. The high pH of root canal sealers may provide several biological advantages. First, high pH of the sealer can promote hard tissue formation such as apical obliteration with calcified tissue [13]. Second, high sealer alkalinity changes the environment in the dentin to a more alkaline pH, possibly interfering with osteoclastic activity and promoting alkalinization in the adjacent tissues, which favors healing [14, 15]. Furthermore, there have been several studies demonstrated that calcium-hydroxide itself inhibited osteoclast activity by various molecular mechanisms [16–19]. Therefore, the high pH of Endoseal may exert an advantageous effect through the aforementioned mechanism compared to conventional resin-based sealers.

In the current study, water solubility of the tested sealers was evaluated because there is a strong link between sealer solubility and periapical reinfection [20]. In our study, the water solubility of Endoseal was the highest among the tested materials although there was no significant difference among the three experimental groups (*P* > 0.05) (Fig. 1c).

Dimensional change demonstrates the shrinkage or expansion of the material after setting. In this study, all the tested materials showed expansion. In previous reports, expansion was also verified for ProRoot and AHplus [21–23]. It is interesting to note that Endoseal expanded significantly more than the other tested materials (*P* < 0.05) (Fig. 1d). Slight expansion may contribute to superior sealing ability, but excessive expansion is undesirable when the material is employed as a root canal filling material as it may elicit cracks in the root [21]. Thus, further tests are required to ascertain if Endoseal effectively seals root canals without increasing the risk of development of cracks or root fracture.

Flow allows a sealer to penetrate into the irregularities and accessory canals of the root canal system [24]. In this study, Endoseal showed significantly higher flow values compared with AHplus (*P* < 0.05) (Fig. 1e). In this respect, Endoseal would have advantage in terms of penetrating into the ramifications and irregularities of root canal system than AHplus. The flow ability is generally influenced by the size of the sealer particles. According to the manufacture, Endoseal contains small particles of calcium-silicate cement to increase the flow. However, if the flow is excessive, the risk of sealer extrusion beyond apical foramen is increased, which could damage periodontal tissues or important anatomical structures such as inferior alveolar nerve or maxillary sinus [25]. Because Endoseal is injectable material which is susceptible to be extruded, clinicians should be careful not to try to fill whole root canal space with it. In this respect, further *in vitro* or *in vivo* study should be performed to conclude the adequate flow of Endoseal.

The addition of radiopaque agents to root canal filling materials should ideally enable their visualization and assessment on a radiograph without altering their chemical properties. According to the ISO standards, root canal sealing materials should be at least 3 mm in aluminum thickness. In the present study, the radiopacity of Endoseal was lower than that of AHplus (*P* < 0.05) (Fig. 2b). However, Endoseal showed much higher radiopacity (over 8 mm/Al) than that required by the ISO standards, similar to ProRoot and AHplus.

Endodontic sealers are often placed in close contact with periapical tissues. Thus, we investigated the biocompatibility of Endoseal in comparison with ProRoot and AHplus. As shown in Fig. 2c, the cell viability was also higher in cells treated with an extract of Endoseal than in cells treated with AHplus on 14-day (*P* < 0.05). However, the cell viability was significantly lower than that of ProRoot. Similarly, SEM observations in this study showed that the cells were attached and had proliferated on the surface of Endoseal and ProRoot, whereas dead cells were found on the surface of AHplus (Fig. 2d–f). These findings indicate that calcium-silicate-based Endoseal has higher biocompatibility compared to epoxy resin-based AHplus and permits adhesion and proliferation of cells.

We also investigated the tissue response to verify whether the materials induce inflammatory reaction *in vivo*. Several *in vivo* studies have shown that most of root canal sealers might induce inflammatory reactions when they contact with connective tissues intimately [26–29]. However, in this study, ProRoot and Endoseal did not show severe inflammatory reaction compared to control group. Calcium-silicate cements such as MTA is believed to induce less inflammation tissue reaction compared to other root canal filling materials [30–34]. In this respect, Endoseal, calcium-silicate cement, might show favorable tissue response comparable to ProRoot although it may contain various chemical ingredients.

We requested the chemical composition of Endoseal from the manufacturer in order to understand in detail the physical properties and biological effects determined in our experiments. According to the manufacturer, Endoseal contains various constituents including hydroxypropyl methylcellulose (HPMC), N-methyl-2-pyrrolidone (NMP), bentonite, bismuth oxide (Bi_2_O_3_), and zirconium oxide (ZrO_2_). HPMC is a non-toxic thickening agent and can react vigorously with oxidizing agents. Use of viscosity agents is suggested for sealer development in order to penetrate into the complex root canal space. NMP is used as a solvent for various chemical agents but has been identified as a toxicant [35]. In this study, Endoseal showed significantly lower cell viability compared to ProRoot (*P* < 0.05) (Fig. 2d), and the presence of NMP in Endoseal might have affected this result. Bentonite is a useful adsorbent of ions in solution as well as fats and oils. It is the main active ingredient of fuller's earth, probably one of the earliest industrial cleaning agents. It is mainly recommended as an ingredient of preparations for dermatologic ointments because its colloidal nature confers detergent properties [36]. Therefore, bentonite is added to the formula to absorb moisture and contamination from the mixture. Bi_2_O_3_ and ZrO_2_ are components in Endoseal that act as radiopacifiers and are widely used in MTA and other endodontic materials [37–39].

## Conclusions

Collectively, the present study indicates that Endoseal has comparable physical properties to MTA, a biocompatible root-end filling material. In addition, Endoseal had favorable biocompatibility/odontogenicity compared to AHplus, a widely used resin-based sealer. Furthermore, this injection-type, self-setting root canal sealer has a clinical advantage in terms of dentist-friendly application. Therefore, within the limitations of this study, we suggest that Endoseal has the potential to be used as a predictable root canal sealer.
